# Importance of Intraoperative Neuromonitoring for Corrective Surgery in Patients with Adolescent Idiopathic Scoliosis

**DOI:** 10.3390/jcm14217693

**Published:** 2025-10-29

**Authors:** Boon Jein Chen, Masato Tanaka, Takeshi Nakagawa, Shinya Arataki, Tadashi Komatsubara, Akiyoshi Miyamoto, Das Gurudip, Maitreya Patil, Koji Uotani, Yoshiaki Oda, Kensuke Shinohara, Tomoyoshi Sakaguchi

**Affiliations:** 1 Department of Orthopaedic Surgery, Okayama Rosai Hospital, Okayama 702-8055, Japan; boonjein2002@hotmail.com (B.J.C.); araoyc@gmail.com (S.A.); t.komatsubara1982@gmail.com (T.K.); hello.akkun.11.136@icloud.com (A.M.); drgurudip@gmail.com (D.G.); maitreyajpatil@gmail.com (M.P.); 2 Department of Central Clinical Laboratory, Okayama Rosai Hospital, Okayama 702-8055, Japan; nakagawa@ms11.megegg.ne.jp; 3 Department of Orthopaedic Surgery, Okayama University Hospital, Okayama 700-8558, Japan; coji.uo@gmail.com (K.U.); odaaaaaaamn@yahoo.co.jp (Y.O.); joker1011ks@yahoo.co.jp (K.S.); 4 Department of Rehabilitation, Okayama Rosai Hospital, Okayama 702-8055, Japan; tomoyoshi0127@gmail.com

**Keywords:** intraoperative neuromonitoring, adolescent idiopathic scoliosis, alarm point, surgical complication

## Abstract

**Background:** Intraoperative neurophysiological monitoring (IONM) has become an invaluable tool for spinal deformity surgery. This study aims to present our experience of using transcranial motor evoked potential (Tc-MEP) as an IONM tool in adolescent idiopathic scoliosis patients undergoing navigation-assisted deformity correction and explore the potential risk factors associated with false-positive and true-positive IONM findings. **Methods:** A retrospective study was conducted in 103 patients (mean age 16.2 ± 4.0 years) undergoing corrective surgery for spinal deformity. All pediatric spinal deformity correction surgeries at the center were performed by a single senior spine surgeon, utilizing navigation and Tc-MEP to enhance the neurological safety profile. The sensitivity and the specificity of Tc-MEP were calculated. **Results:** Of the total cases, 87 patients (84.5%) exhibited no IONM signal alert and did not experience any postoperative neurological deficits, representing true negatives. There were no false-negative cases, which gives a negative predictive value of 100%. Significant IONM signal alerts were observed in 16 patients (15.5%), but only two patients (1.9%) experienced a postoperative motor deficit, representing true positives, which yielded a positive predictive value of 12.5%. **Conclusions:** This study demonstrated the sensitivity and specificity of Tc-MEP to be 100% and 86.3%, respectively, with a false-positive rate of 13.7%. Blood loss was the only factor significantly associated with IONM alerts, while age, gender, surgical duration, and anesthetic modality showed no significant differences.

## 1. Introduction

Although most patients with adolescent idiopathic scoliosis are asymptomatic, severe spinal deformities can lead to compromised cardiopulmonary function and psychological issues related to appearance, such as lower self-esteem, anxiety, and depression, which may necessitate corrective surgery. However, any neurological complications resulting from the corrective procedure can have disastrous consequences.

The spinal cord can be compromised during spinal surgeries either from direct mechanical forces on the cord by hardware or due to ischemia sustained during intraoperative corrective maneuvers or compromised cord perfusion [1]. Intraoperative neuromonitoring (IONM) has become an invaluable tool for monitoring and assessing spinal cord integrity during spinal surgery, with the aim of mitigating neurological injury and improving surgical outcomes [2]. Since the introduction of spinal cord evoked potential (SCEP) in 1972 [3], various IONM techniques have been developed over the past decades to alert the surgical team about impending neurological injury [3,4]. These techniques include somatosensory evoked potentials (SSEP) [2], motor evoked potentials (MEP) [2], and electromyography (EMG) [5], which are either used in isolation or in combination. The IONM techniques most frequently employed during spinal surgery are SSEP and transcranial MEP.

SSEPs have gained widespread adoption progressively since the 1970s to monitor the status of the dorsal column-medial lemniscus pathway intraoperatively [6,7]. However, the major drawbacks of SSEP are its inability to monitor the anterior and lateral spinal cord for injury or ischemia, resulting in significant false-negative rates [8,9,10]. The inherent delay in feedback resulting from the need to average responses to provide results is also an essential disadvantage of this IONM modality [11].

MEP was first introduced in 1980 by Merton and Morton to overcome the limitations of SSEP in intraoperative monitoring of neurological deficits [12]. MEP assesses the anterior corticospinal tract, which is more sensitive to ischemic changes [13], especially during correction maneuvers in pediatric spinal deformity surgery, which may compromise anterior spinal artery blood flow [1]. Transcranial MEP (Tc-MEP) stimulates the motor cortex to elicit a motor response in the upper and lower limbs, which is picked up and recorded by surface recording electrodes placed on the respective muscles [11]. Despite MEPs being only able to monitor the status and integrity of the corticospinal tract in the anterior spinal cord, MEP was reported to have higher sensitivity but lower specificity than SSEP, with lower false-negative rates in identifying intraoperative spinal cord injury during corrective surgery for adolescent idiopathic scoliosis (AIS), in contrast to the lower sensitivity but higher specificity of SSEP [14]. However, Tc-MEP is not without its own specific limitations, which include the lack of consensus on alert criteria, its high sensitivity to anesthesia, and a reported high false-positive rate [14,15,16,17].

Few studies have evaluated the sensitivity and specificity of IONM in adolescent idiopathic scoliosis (AIS) surgery [14]. This study aims to present our experience with MEP in AIS patients undergoing navigation-assisted deformity correction and explore the potential risk factors associated with false-positive and true-positive IONM findings.

## 2. Materials and Methods

This study was designed as a retrospective single-center observational study based on intraoperative neuromonitoring and clinical data in deformity surgery. This study was approved by the Institutional Review Board of Okayama Rosai Hospital (research approval no. 568), and the study’s protocol adhered to the principles outlined in the Declaration of Helsinki. The necessary informed consents were duly signed and obtained from all the patients involved in this study.

### 2.1. Patient Selection

**Figure 1:**
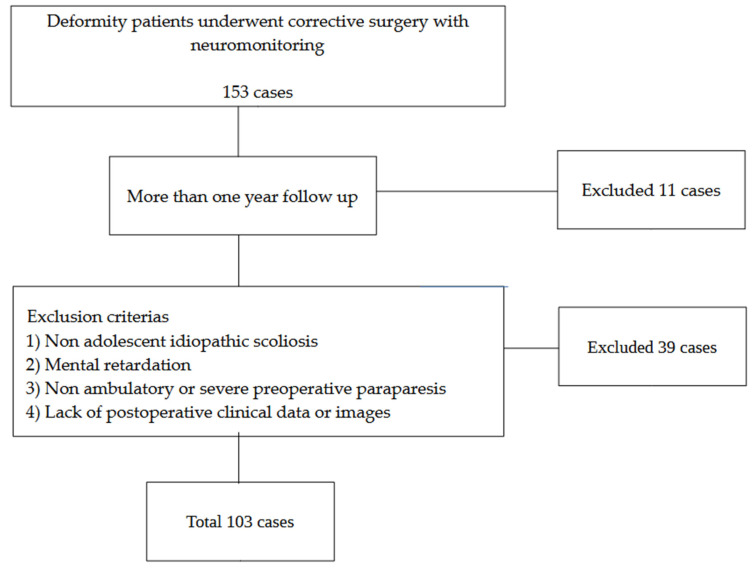
Patient selection.

The information of all patients who underwent deformity correction surgeries for adolescent idiopathic scoliosis between May 2017 and March 2025 was extracted from the institution’s digital medical records and analyzed. Inclusion criteria: (1) deformity correction with neuromonitoring and (2) more than one year of follow-up. Exclusion criteria: (1) non-idiopathic scoliosis, (2) mental retardation, (3) non-ambulatory or severe preoperative paralysis, and (4) lack of postoperative images or clinical data. A review of medical records identified 153 patients (117 females and 36 males; mean age, 19.3 years) who met the inclusion criteria. Of these, 38 patients were excluded as they met one or more exclusion criteria, and 11 patients declined to provide consent for participation or less than one year follow-up, resulting in their exclusion from the study (Figure 1).

### 2.2. Neuromonitoring Technique

**Figure 2:**
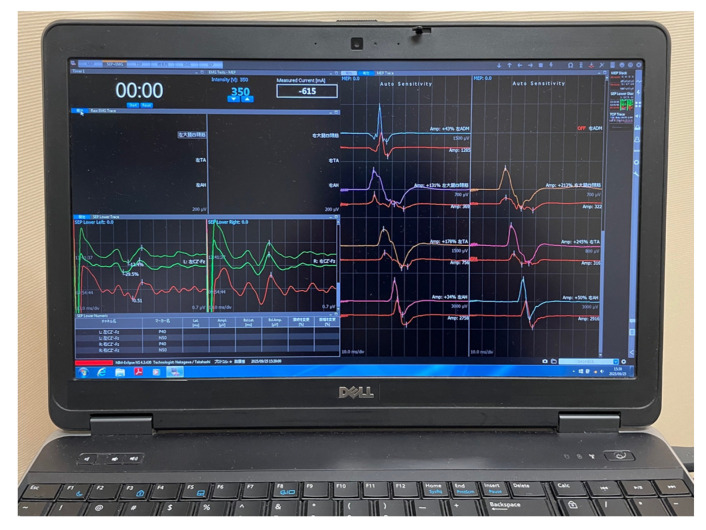
Medtronic Nim Eclipse IONM system. (Picture of Medtronic Nim Eclipse).

All spinal deformity correction surgeries in the center were performed by a single senior spine surgeon with the aid of navigation and IONM to enhance neurological safety profile. The IONM technique routinely employed at our center is transcranial motor evoked potentials (Tc-MEP) combined with EEG monitoring. Transcranial electrical stimulation was performed using a Medtronic Nim Eclipse IONM system (Medtronic, Minneapolis, MN, USA) (Figure 2).

Corkscrew stimulating electrodes were placed on the scalp at C3 and C4, as well as 6 cm anterior to Cz, in accordance with the international 10–20 system, to target the motor cortex. MEP recording electrodes were placed over the trapezius and abductor pollicis brevis in the unilateral upper extremity as a control, and the quadriceps, tibialis anterior, and abductor hallucis longus muscles in the lower extremities bilaterally. Transcranial stimulation was administered in trains consisting of five pulses at 200–600 V, 100 mA, each lasting 250 µs, with an interstimulus interval of 1 ms. Recordings were made over a 100 ms period, with a minimum interval of 30 s between successive stimulus trains. Monitoring was conducted at 20 min intervals, with additional stimulation administered after each pedicle screw insertion, following correction maneuvers, and upon the surgeon’s request for further measurements. Postoperative motor deficit is defined as the muscle strength that decreased by at least one grade on the manual muscle test. Neurological function is examined in the recovery room and daily until discharge.

Baseline MEP is always recorded for reference after anesthetic induction, and prior to the transfer and post-positioning of the patient on the operating table. In the event of the presence of an IONM alert, the surgical and anesthetic team will be notified immediately. The MEP alert cutoff point is defined as a unilateral or bilateral reduction of at least 50% from baseline amplitude, which signals motor pathway compromise with potential neurological deficit. The equipment will be checked for malfunction, e.g., loosening of the electrode. The surgical procedure will be stopped immediately with reversal of surgical manipulation and surgical strategy modified as needed. If the amplitude reduction occurs following screw insertion, screw positions will be verified intraoperatively using an O-arm. Similarly, any corrective maneuvers (e.g., rod removal) will be reversed if a TcMEP decrease is observed during correction. Rescue intervention, such as warm saline irrigation of the spinal cord and intravenous steroid injection, will be promptly carried out. The patient’s temperature, blood pressure, oxygenation, and hemoglobin level will also be optimized accordingly. In the event of a persistent reduction in TcMEP amplitude despite appropriate interventions, Stagnara wake up test will be performed to assess the patient’s neurological function. All remaining pedicle screws will be inserted according to preoperative planning without rod application. Neurological (motor and sensory) examination was performed immediately post-extubation when the patient regained full GCS and daily thereafter until discharge from hospital. New onset of neurological deficit is defined as a decline of more than one motor power grade post-surgery. Absence of motor recovery by three months after index surgery is classified as permanent, while the recovery of motor power within three months is classified as temporary. Rescue cases are defined as those in which waveform recovery was achieved after interventions in response to alerts and no postoperative deficit.

### 2.3. Definitions

#### 2.3.1. Intraoperative Neuromonitoring Alert Definition

Intraoperative neuromonitoring alert is defined as a unilateral or bilateral reduction in Tc-MEP signal amplitude by at least 50%.

#### 2.3.2. True Positive Definition

A true-positive IONM alert is defined as a sustained, irreversible decrease in signal amplitude, despite rescue interventions, and is associated with a postoperative neurological deficit.

#### 2.3.3. False Positive Definition

A false-positive IONM alert is defined as a significant and sustained decrease in signal amplitude that persists despite rescue interventions, but is not associated with any postoperative neurological deficit.

#### 2.3.4. True Negative Definition

A true-negative IONM alert is defined as no IONM alert and is not associated with any postoperative neurological deficit.

#### 2.3.5. False Negative Definition

A false-negative IONM alert is defined as the absence of an IONM alert, despite the patient experiencing a neurological deficit in the postoperative period.

#### 2.3.6. Sensitivity and Specificity

Sensitivity = True positive/(True positive + False negative) × 100

Specificity = True negative/(True negative + False positive) × 100

### 2.4. Anesthetic Protocol

Most of the patients underwent anesthetic induction and maintenance protocol using total intravenous anesthesia (TIVA); however, 18 patients received inhalational anesthesia at the anesthesiologist’s discretion.

### 2.5. Statistical Analysis

Based on a reduction in Tc-MEP amplitude to less than 50% of the baseline value, patients were divided into IONM alert-positive and negative groups. Results are presented as numbers and percentages or means ± standard deviations. Statistical analysis of categorical factors such as gender and mode of anesthesia was performed using the chi-square test. The statistical significance of non-categorical data, such as age, blood loss, duration of surgery, and number of fusion levels, was determined by the Mann–Whitney U test. Differences were considered significant at a *p*-value of <0.05.

## 3. Results

### Patient Demographics

The study involved a total of 103 patients (84 female and 19 male), with an average age of 16.2 years at the time of surgery. 16 patients (14 females and 2 males) had a positive IONM alert, while the remaining 87 patients (70 females and 17 males) were negative.

Patients were categorized into two cohorts according to the presence or absence of IONM alerts. Demographic as well as perioperative variables—including age, gender, anesthetic modality, operative duration, number of fused levels, and intraoperative blood loss—were compared with each other (Table 1) and subjected to statistical analysis (Table 2) to assess for significance.

**Figure 3:**
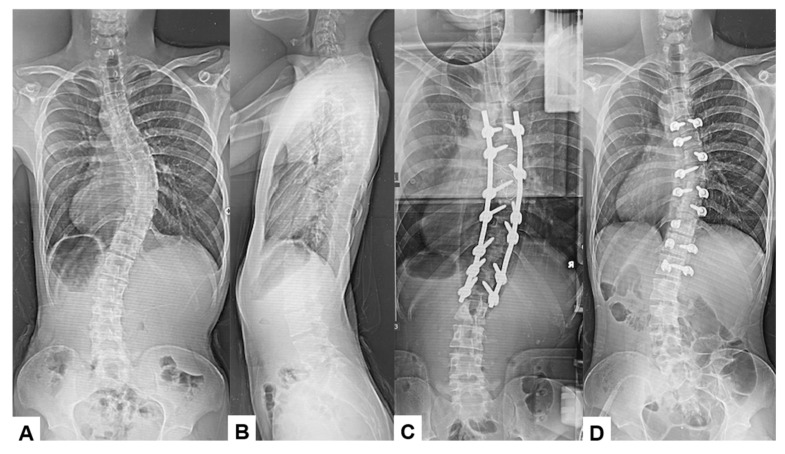
True TcMEP-positive case. 18-year-old male, AIS, T5-L1 corrective fusion. (**A**) Preoperative posteroanterior radiogram, (**B**) preoperative lateral radiogram, (**C**) intraoperative posteroanterior radiogram prior removal of rods, and (**D**) intraoperative posteroanterior radiogram post removal of rods.

**Figure 4:**
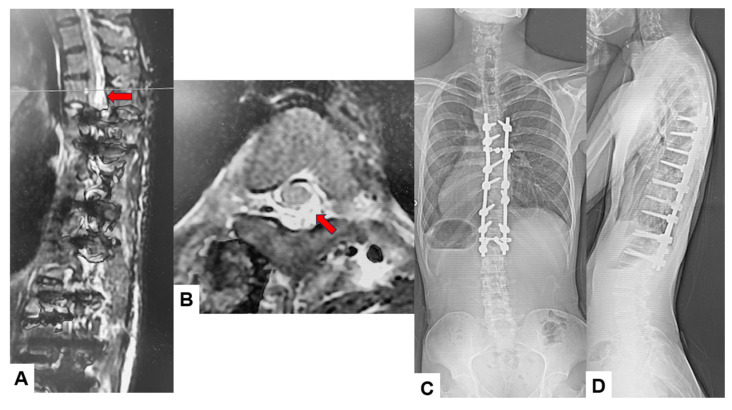
True TcMEP-positive case. 18-year-old male, AIS, Postoperative MRI and revision surgery. Red arrows indicate an acute spinal epidural hematoma. (**A**) Postoperative T2-weighted sagittal MR imaging, (**B**) postoperative T2-weighted axial MR imaging at T4, (**C**) final posteroanterior radiogram, and (**D**) final lateral radiogram.

Among the 16 patients who experienced significant IONM alerts, only two (12.5%) developed postoperative neurological deficits, representing true-positive cases. The first case involved an 18-year-old male who underwent an eight-level fusion procedure (T5-L1) and exhibited a Tc-MEP alert following a substantial epidural hemorrhage. IONM alert persisted despite anesthetic rescue interventions. Hence, the corrective maneuver was reversed with the removal of rods. A wake-up test performed confirmed the presence of neurological deficits. The total intraoperative blood loss was 4,500 mL, and the operative time was 391 min. The patient awoke with paraparesis (American Spine Injury Association Score C) but achieved complete neurological recovery within seven months (Figure 3 and Figure 4). The cause of this patient’s paraparesis might be due to spinal ischemia or epidural hematoma. The second case involved an 18-year-old female who underwent an 11-level fusion from T5 to L3. An IONM alert was detected during deformity correction, with a total operative duration of 245 min and intraoperative blood loss of 1380 mL. The patient (American Spine Injury Association Score C) achieved complete neurological recovery by 3 months postoperatively. The cause of this patient’s paraparesis was unclear.

The remaining 14 patients with persistent IONM signal changes did not have postoperative neurological deficits, representing false positives. There were no false-negative cases in our study. The test demonstrated an overall sensitivity of 100% and a specificity of 86.3%, with a false-positive rate of 13.7%.

The amount of blood loss was the only significant factor for having a positive TcMEP alert. At the same time, age, gender, duration of surgery, and mode of anesthesia were not found to be statistically different between the two groups. The results of the comparisons were summarized in Table 1. The diagnostic accuracy evaluation results of Tc-MEP are shown in Table 2.

## 4. Discussion

There is a paucity of literature specifically evaluating the sensitivity and specificity of motor evoked potential (MEP) in adolescent idiopathic scoliosis (AIS) surgery [14,18,19,20,21]. In the limited available studies, MEP has demonstrated high diagnostic performance, with reported sensitivity and specificity reaching up to 100% and 96%, respectively [14,22,23]. Despite its high sensitivity, MEP is susceptible to intraoperative factors such as hypotension and spinal cord ischemia. This vulnerability may be attributed to the tenuous blood supply of the anterior spinal cord, which relies predominantly on the anterior spinal artery supplied by the radicular arteries [24,25]. Given this inherent sensitivity to ischemic changes, MEP is particularly well-suited for intraoperative monitoring of spinal cord integrity during deformity correction procedures in AIS [5].

In our study, there was no false-negative intraoperative neuromonitoring (IONM) alert. The high sensitivity of transcranial MEP (Tc-MEP) monitoring likely contributed to the absence of false-negative cases observed in this study. Modi et al. reported the only known case of a false-negative MEP in a 15-year-old girl with severe kyphoscoliosis secondary to neurofibromatosis, which was postulated to result from intraoperative hypotension or excessive blood loss [26].

Only two true-positive cases were identified in this study: one occurred during deformity correction maneuvers, while the other case was related to excessive blood loss. Importantly, none of the IONM alerts or neurological complications in our study were attributable to pedicle screw malposition. This can be attributed to the high accuracy achieved with navigation-guided screw placement. Additionally, the availability of intraoperative CT imaging with the O-arm system enabled immediate verification of screw positioning in cases of doubt.

Our study also identified 14 cases (13.7%) of false-positive alerts during the correction of AIS surgery. These findings are comparable to previously published reports, with incidences ranging from as low as 0.014% [27] to 13% [16]. Multiple hypotheses have been proposed in the literature regarding the causes of false-positive MEP alerts, including the use of inhalational anesthetics, prolonged surgical duration, inadequate adjustment of the anesthetic regimen for fade [16,28], anesthetic depth [29,30], preexisting motor deficit [17], patient obesity [31], greater lability in mean arterial pressure [20], blood loss or hypotension [20], and the absence of standardized alarm criteria (lower threshold) for MEP monitoring [32].

Our study has found blood loss to be the only statistically significant factor associated with positive IONM alert. The positive IONM alert group had a mean blood loss of 1561 mL compared to 1072 mL in the negative IONM alert group (*p* < 0.05). Hung et al. [33] also reported similar findings of blood loss being the only significant factor associated with IONM loss in their study of spine deformity correction surgery. Significant blood loss in spinal deformity correction surgery may lead to a low intraoperative hemoglobin level, which may result in reduced spinal cord perfusion with subsequent cord ischemia, causing loss of or reduced MEP signal [34].

Although the mean surgical time of the patients in the positive IONM alert group (296.5 min) in our study is greater than those in the negative IONM alert group (272.2 min), the difference did not achieve statistical significance. Anesthetic fade has been reported in the literature to affect IONM signals. Anesthetic fade describes the progressive decline in the amplitude of MEP during surgery, resulting from the effects of anesthetic agents on the central nervous system and neuromuscular transmission [16,28]. MEP is particularly sensitive to volatile anesthetics and high-dose intravenous agents, such as propofol. Prolonged or continuous anesthetic administration can lead to a gradual reduction in MEP amplitude (anesthetic fade), which may be further exacerbated by extended surgical duration and significant intraoperative blood loss [16]. The mean duration of pediatric scoliosis procedures in this study exceeded 270 min, rendering them potentially susceptible to anesthetic fade. Furthermore, as anesthetic fade is dose-dependent, anesthetic depth—which can be indirectly monitored using electroencephalography—should be taken into account when interpreting MEP changes [28,35]. Despite the association of high concentration inhalational anesthesia with false-positive Tc-MEP alerts, this association is not statistically significant (*p* = 0.389) in this study, possibly due to the small number of positive alerts (*n* = 16), which limits statistical power.

One of the two true-positive IONM alert patients in our study sustained spinal cord injury during deformity correction maneuver. As spinal correction maneuvers, which carry the most significant risk of cord injury, are always performed in the later stages of surgery, the effects of anesthetic fade on MEP may be further amplified [16,36,37]. Lee et al. reported preoperative Cobb angle of the major structural curve and number of levels fused as risk factors for positive IONM alert during AIS deformity correction surgery [37]. However, in our study, the number of levels fused was not found to be a significant factor.

The absence of a standardized cutoff for MEP amplitude reduction may also contribute to false-positive alerts, as there is no consensus regarding what constitutes a clinically significant change. Consequently, different institutions and surgeons often adopt variable, empirically derived thresholds. This lack of uniformity increases the likelihood that intraoperative physiological fluctuations may be misinterpreted as meeting alert criteria, thereby generating false-positive alarms. We adopted a 50% decrease in amplitude from baseline as the MEP alert cut-off point, considering that lower thresholds for MEP amplitude reduction are known to increase the sensitivity of intraoperative monitoring. However, this lower threshold is also associated with a higher rate of false-positive alerts. While completely eliminating false-positive cases is impractical, as it would require extremely stringent alert criteria and, thus, may compromise sensitivity, certain measures can help minimize their occurrence. Reducing false-positive Tc-MEP alerts in AIS surgery necessitates careful control of anesthetic depth and physiological parameters (maintaining the mean arterial pressure) and the use of standardized alert thresholds [16,20,28,29,30,32]. Incorporating multimodal intraoperative neurophysiological monitoring (IONM), such as combining Tc-MEP with EMG, may further reduce false-positive rates [38]. Although the identification of false positives intraoperatively is not always straightforward, it may be facilitated by a lack of concordance with other IONM modalities or by transient changes in MEP signals.

It is essential to identify and minimize false-positive MEP alerts, as the failure to do so may lead to unnecessary interventions. Anesthetic fade can often be distinguished from true neurological injury by its gradual onset, in contrast to the abrupt loss of MEP seen with actual cord compromise [39]. Strategies to address anesthetic fade include adjusting the propofol dosage to reverse suppression and increasing stimulation intensity to assess potential MEP recovery. Incorporating multimodal monitoring can help minimize false-positive MEP findings. The use of upper limb recordings as a control can also aid in differentiating true from false MEP changes [39,40]. Concurrent amplitude reduction in both upper and lower limb or bilateral MEPs is more indicative of systemic factors, such as hypotension, anemia, or reduced hematocrit, rather than spinal cord injury [40].

This study has several limitations. First, it was a retrospective analysis conducted at a single center by a single surgeon, which may limit the generalizability of the findings. Second, the absence of a universal consensus on what constitutes a significant change in MEP introduces variability in the interpretation of alerts, thereby influencing the reported sensitivity and specificity. Third, neuromonitoring outcomes are susceptible to various factors such as anesthetic regimen, physiological parameters, and the technical expertise of the monitoring team, all of which may differ across institutions and further limit external applicability. Future multicenter, prospective studies with larger patient cohorts and standardized anesthetic protocols and monitoring criteria are needed to more accurately define the diagnostic accuracy and clinical utility of intraoperative neuromonitoring in AIS corrective surgery.

## 5. Conclusions

Tc-MEP is a valuable intraoperative neuromonitoring modality that contributes to the prevention of neurological complications during AIS corrective surgery. This study demonstrated high sensitivity (100%) and reasonable specificity (86.3%) and a false-positive rate of 13.7%, which is consistent with previously published reports. Blood loss is the only significant factor associated with a positive IONM alert, whereas age, gender, duration of surgery, and anesthetic modality are not.

## Figures and Tables

**Table 1:** Comparison of parameters between IONM alert-positive and negative groups.

	Positive IONM Alert	Negative IONM Alert	*p* Value
Male	2	17	0.516
Female	14	70	
Age (years)	16.2 ± 4.8	16.2 ± 3.9	0.487
Duration of surgery (minutes)	296.5 ± 56.5	272.2 ± 64.5	0.211
Blood loss (mL)	1560.6 ± 1066.9	1071.6 ± 599.2	0.028 *
Level of fusion	10.2 ± 3.0	9.7 ± 3.3	0.478
Inhalational anesthesia	4	14	0.389
TIVA	12	73	

IONM: Intraoperative Neuromonitoring, TIVA: Total intravenous anesthesia. * *p* < 0.05.

**Table 2:** Results of the intraoperative neurophysiological monitoring techniques.

	IONM Alert Positive	IONN Alert Negative
Neurological deficit present	2 (true positive)	0 (false negative)
No neurological deficit	14 (false positive)	87 (true negative)
Sensitivity	100%	
Specificity	86.1%	
Positive predictive value	12.5%	
Negative predictive value	100%	
False positive	13.9%	
False negative	0%	

IONM: Intraoperative Neuromonitoring.

## Data Availability

The data presented in this study are available in the article.
